# Factors associated with foot self care behavior among older adults with recurrent diabetic foot ulcer: a cross-sectional study

**DOI:** 10.3389/fendo.2026.1856183

**Published:** 2026-06-03

**Authors:** Liping Lin, Jianbing He, Hongjian Zhang, Aiwen Yang, Qiuni Cai

**Affiliations:** 1 Department of Vascular Surgery, Zhongshan Hospital of Xiamen University, School of Medicine, Xiamen University, Xiamen, China; 2 Department of Thoracic Surgery, Zhongshan Hospital of Xiamen University, School of Medicine, Xiamen University, Xiamen, China; 3 Department of Medical Oncology, Zhongshan Hospital of Xiamen University, School of Medicine, Xiamen University, Xiamen, China; 4 Department of General Surgery, Zhongshan Hospital of Xiamen University, School of Medicine, Xiamen University, Xiamen, China

**Keywords:** aged, cross-sectional studies, diabetes mellitus, diabetic foot, health behavior, recurrence, self-care

## Abstract

**Background:**

Diabetic foot ulcers (DFU) are a severe complication of diabetes, with high recurrence rates and substantial clinical burden. Foot self-care behavior is essential for preventing ulcer recurrence, yet evidence specifically targeting older adults with recurrent DFU remains limited. This study aims to identify the factors associated with foot self-care behavior among older adults with recurrent diabetic foot ulcers.

**Methods:**

A cross-sectional study was conducted using a purposive sampling technique among 170 older adults (≥60 years) with recurrent DFU recruited from the multidisciplinary diabetic foot clinics of three general hospitals in China between January 2023 and December 2024. The study adhered to the STROBE guidelines for cross-sectional studies. Data were collected using structured questionnaires, including the Nottingham Foot Care Assessment (NAFF), Foot Care Confidence Scale, Multidimensional Scale of Perceived Social Support, and Geriatric Depression Scale (GDS-15). Clinical characteristics were extracted from medical records. Multiple linear regression analysis was performed to identify factors associated with foot self-care behavior.

**Results:**

The mean total NAFF score was 48.5 ± 10.2, indicating a moderate level of foot self-care. Foot care self-efficacy (β = 0.41, P < 0.001), receipt of foot care health education (β = 0.25, P = 0.001), perceived social support (β = 0.19, P = 0.008), and depressive symptoms (β = −0.17, P = 0.021) were factors associated with foot self-care behavior, collectively explaining 47.2% of the variance in foot self-care behavior (adjusted R² = 0.472).

**Conclusion:**

Foot self-care behavior among older adults with recurrent DFU is moderate and is associated with modifiable psychosocial factors. Interventions targeting self-efficacy, health education, social support, and depressive symptoms may contribute to improved self-care and potentially reduce ulcer recurrence. Longitudinal and interventional studies are warranted to confirm these associations and evaluate clinical effectiveness.

## 1 Introduction

Diabetes mellitus (DM) is a major global health challenge, with its prevalence projected to increase from 10.5% (536.6 million people) in 2021 to 12.2% (783.2 million people) by 2045, and its impact is particularly significant in the elderly population (1, 2). Diabetic foot ulcer (DFU) is one of the most severe complications, affecting approximately 18.6 million people worldwide annually (3, 4). DFU leads to serious clinical outcomes, including high rates of infection, amputation, and mortality (3). Crucially, the ulcer recurrence rate is extremely high, with approximately 40% of patients experiencing recurrence within 1 year and 65% within 5 years (5).

Foot self-care behavior is a core measure for preventing DFU and reducing recurrence rates (6). Despite this, patient adherence remains suboptimal, which directly contributes to ulcer recurrence (7–9). Factors associated with self-care include perceived family support, diabetes-related education, and self-care confidence (6, 10). However, patients with DFU generally exhibit low health literacy, and the cognitive decline frequently observed in the elderly population poses a significant barrier to effective preventive self-care (11, 12).

Crucially, research specifically targeting older adults with recurrent DFU is extremely limited (13). This population is uniquely challenged by advanced age, multimorbidity, and the cumulative physical and psychological burden of repeated ulcer trauma, which may profoundly affect their self-care behaviors. Therefore, this study aimed to identify the factors associated with foot self-care behavior among older adults with recurrent diabetic foot ulcer. By analyzing demographic, disease-related, and psychosocial variables, the findings are expected to provide a basis for developing targeted nursing interventions.

## 2 Methods

### 2.1 Study design and setting

This cross-sectional study was conducted between January 2023 and December 2025 at the multidisciplinary diabetic foot clinics of three general hospitals in China. The study adhered to the Strengthening the Reporting of Observational Studies in Epidemiology (STROBE) guidelines for cross-sectional studies (14). The study focused on older adults with recurrent DFU. Recurrent DFU was defined as the occurrence of a new foot ulcer at the same or a different site after complete healing of a previous ulcer, consistent with previous definitions and international guidelines (15).

### 2.2 Participants

#### 2.2.1 Inclusion and exclusion criteria

Participants were eligible if they met the following criteria:

1. aged 60 years or older;
2. had a confirmed diagnosis of diabetes mellitus;
3. had a physician-confirmed recurrent DFU at the time of recruitment;
4. provided written informed consent.

Patients were excluded if they:

1. had their first episode of DFU;
2. had a diagnosed severe psychiatric disorder and cognitive impairment;
3. were critically ill or hemodynamically unstable;
4. had major lower-limb amputation above the ankle or any condition that precluded participation in the interview.
5. refused or were unable to provide informed consent.

A purposive sampling technique was employed to consecutively recruit eligible participants from the study sites to ensure a representative sample of the target population.

#### 2.2.2 Sample size

The required sample size was estimated using G*Power version 3.1. Based on Cohen’s conventions for behavioral and health research (16) and prior related studies (6, 10), a medium effect size (f² = 0.15) was specified for the multiple linear regression model, with a significance level of 0.05, a statistical power of 0.80, and 15 potential associated factors. The minimum required sample size was 139. Final recruitment yielded 170 participants. All participants signed informed consent and completed the study without dropout, satisfying the minimum sample size requirement.

### 2.3 Data collection instruments

#### 2.3.1 Sociodemographic and clinical characteristics

A structured questionnaire was employed to collect sociodemographic information from the participants, including age, gender, marital status, educational level, and place of residence (rural, urban). Clinical characteristics were obtained through participant interviews and medical record reviews, encompassing diabetes duration, current diabetes treatment modalities (no medication, oral medication only, insulin injection only, oral medication combined with insulin injection), glycated hemoglobin (HbA1c), body mass index (BMI), smoking history, alcohol consumption history, diabetic peripheral neuropathy, hypertension, hyperlipidemia, and whether the participant had received foot care health education. Ulcer severity was assessed according to the Wagner classification system (17).

#### 2.3.2 Foot self-care behavior

The Nottingham Foot Care Assessment (NAFF) was originally developed by Lincoln and colleagues, and later sinicized by Chinese scholars Li et al. (18) to evaluate patients’ foot self-care behaviors. The scale consists of five dimensions and 24 items, including foot inspection (3 items), foot cleaning (4 items), foot protection (5 items), footwear and sock selection (9 items), and healthcare-seeking behavior (3 items). A Likert 4-point scoring method is adopted, with total scores ranging from 0 to 72. The Cronbach’s α coefficient of the scale is 0.77, and the test-retest reliability is 0.76.

#### 2.3.3 Foot care self-efficacy

Participants’ confidence in performing recommended foot care activities was measured using the Foot Care Confidence Scale (19). This 12−item, unidimensional self−report instrument assesses perceived ability to carry out essential foot self−care tasks. Each item is rated on a 5−point Likert scale, yielding a total score ranging from 12 to 60, with higher scores indicating greater foot care self−efficacy. In this study, the Cronbach’s α coefficient was 0.92.

#### 2.3.4 Perceived social support

Perceived social support was measured using the Multidimensional Scale of Perceived Social Support (MSPSS) (20). This 12-item scale evaluates support from three sources: family (4 items), friends (4 items), and significant others (4 items). Items are rated on a 7-point Likert scale. Higher scores indicate stronger perceived social support. The Cronbach’s α coefficient in this study was 0.877.

#### 2.3.5 Depressive symptoms

Depressive symptoms were assessed using the 15-item Geriatric Depression Scale (GDS-15), which is widely used in older populations (21). Each item is answered in a “yes/no” format. Higher scores indicate more severe depressive symptoms. The Cronbach’s α coefficient of the scale is 0.909.

### 2.4 Data collection procedure

Eligible participants were screened and identified by clinicians or wound care nurses. After eligibility screening, professionally trained research assistants approached potential participants to explain the study purpose and obtained written informed consent. To minimize interviewer bias, research assistants utilized a structured interview script with standardized prompts for each question. Inter-rater reliability was not formally assessed; however, all assistants underwent a full-day training session focused on standardized administration. Considering that some elderly individuals may have visual impairments, low literacy, or physical discomfort related to diabetic foot ulcers, the researchers assisted participants in completing questionnaires through face-to-face interviews in a quiet area of the clinic or ward to collect data. Each interview lasted approximately 20 to 30 minutes. The data collection process was scheduled to not interfere with the patients’ clinical care routines. Administrative approvals were obtained from all three participating hospitals before data collection commenced, and the study was approved by the Human Ethics Committee of Zhongshan Hospital of Xiamen University (Approval No. 2022-085). Participant confidentiality was ensured by de-identifying all data and restricting access to the research team.

### 2.5 Statistical analysis

Data analysis was performed using IBM SPSS Statistics version 23.0. All variables were analysed using descriptive statistics. The level of self-care behaviours according to demographic, disease-related and laboratory characteristics was analysed using an independent t test, ANOVA and Pearson’s correlation.

Multiple linear regression analysis was conducted with the total score of foot self-care behaviors as the dependent variable to identify factors associated with foot self-care behavior. Variables with a *P*-value < 0.05 in the univariate analysis were included in the multivariate model. Prior to interpreting the model, regression assumptions were tested, including linearity, independence of errors, homoscedasticity, normality of residuals, and multicollinearity. Multicollinearity was assessed using variance inflation factor (VIF) and tolerance values. A two-sided *P*-value < 0.05 was considered statistically significant. Additionally, *post-hoc* subgroup analyses were performed to explore the consistency of the identified associations across key clinical strata. Participants were dichotomized by age (<70 years vs. ≥70 years) and glycemic control level (HbA1c <7.5% vs. ≥7.5%). Interaction terms between each stratifying variable and the significant associated factors from the main model were tested in separate regression models.

### 2.6 Ethical considerations

This study was approved by the Human Ethics Committee of Zhongshan Hospital of Xiamen University (Approval No. 2022-085). All procedures were conducted in accordance with the Declaration of Helsinki. Written informed consent was obtained from all participants before enrollment. Participants were informed that their participation was voluntary, that they could withdraw at any time without affecting their treatment, and that all collected data would be kept confidential and used only for research purposes.

## 3 Results

### 3.1 Participant characteristics

**Figure 1:**
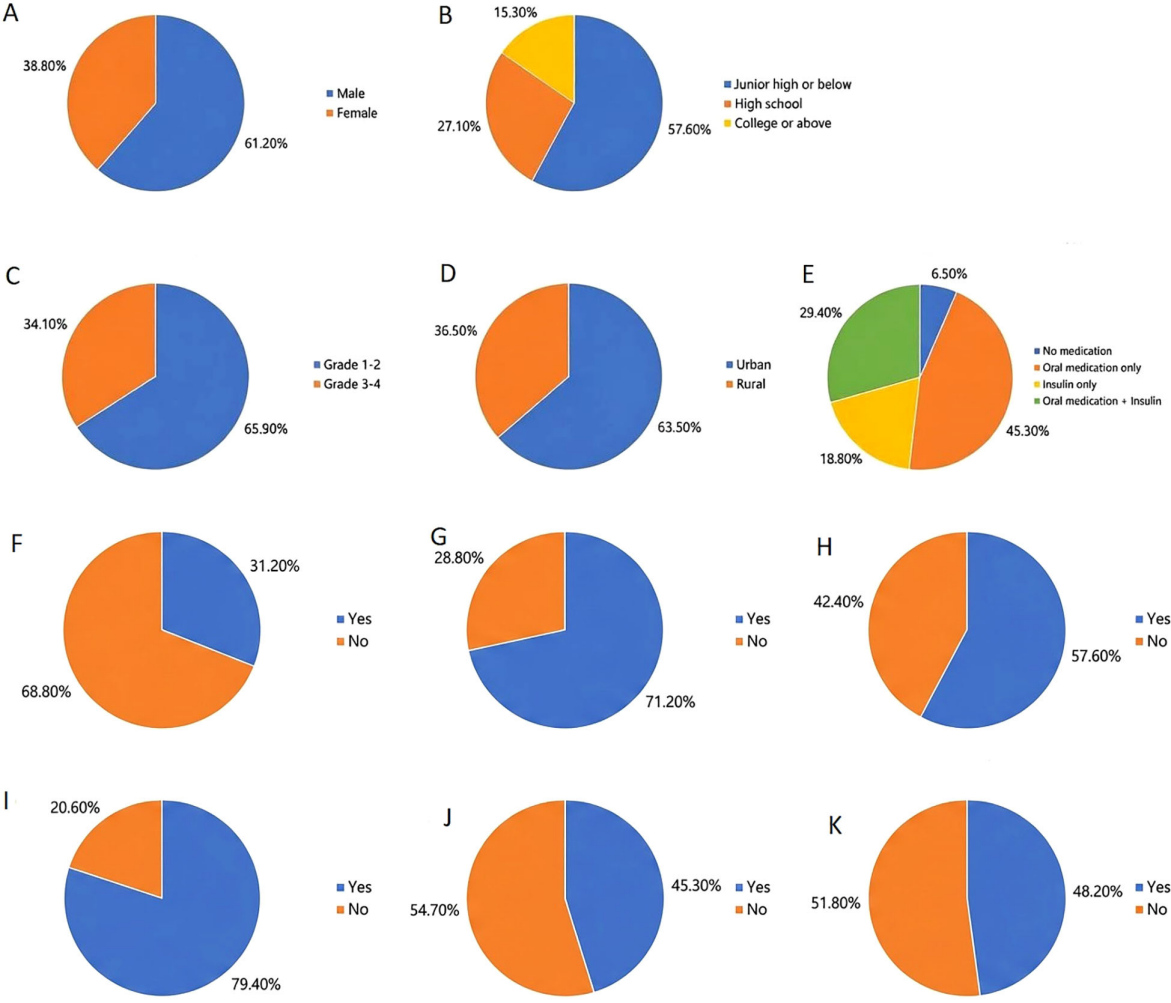
Pie chart of participant characteristics. **(A)** Gender; **(B)** Education level; **(C)** Wagner Grade; **(D)** Residence; **(E)** Diabetes Treatment Regimen; **(F)** Alcohol consumption; **(G)** Hypertension; **(H)** Hyperlipidemia; **(I)** Diabetic peripheral neuropathy; **(J)** Received foot care health education; **(K)** Smoking history.

A total of 170 elderly patients with recurrent diabetic foot ulcers were enrolled in this study. The age range was 60 to 85 years, with a mean age of (68.4 ± 5.6) years. Among them, 104 (61.2%) were male and 66 (38.8%) were female. The mean duration of diabetes was (15.2 ± 7.1) years, and the mean BMI was (24.1 ± 3.2) kg/m^2^. Regarding clinical characteristics, the mean HbA1c level was (8.3 ± 1.6)%. A history of smoking was reported by 82 participants (48.2%), and a history of alcohol consumption was reported by 53 participants (31.2%). Hypertension was present in 121 patients (71.2%), and hyperlipidemia was present in 98 patients (57.6%). Among the current diabetes treatment plans, oral medication alone was the most common regimen, accounting for 45.3% of cases. The participant characteristics are presented in **Figure 1**.

### 3.2 Scores of the scales

The total score of foot self-care behaviors (NAFF) for the 170 patients was (48.5 ± 10.2) points, indicating a moderate level. The scores for each dimension are detailed in **Table 1**. Scores of the psychosocial-related scales were as follows: foot care self-efficacy scored (32.4 ± 7.8) points, perceived social support scored (58.6 ± 12.4) points, and depressive symptoms (GDS-15) scored (6.1 ± 3.5) points.

**Table 1:** Descriptive statistics of foot self-care behavior and psychosocial measures (N = 170).

Variable	Actual range	Mean ± SD
NAFF Foot Self-Care Behavior Total Score	22 – 69	48.5 ± 10.2
Foot inspection (3 items)	0 – 9	5.2 ± 1.8
Foot cleaning (4 items)	4 – 12	11.1 ± 2.1
Foot protection (5 items)	2 – 15	10.8 ± 3.2
Footwear and sock selection (9 items)	5 – 27	17.2 ± 4.5
Healthcare-seeking behavior (3 items)	0 – 9	4.2 ± 1.9
Foot Care Self-Efficacy Score	14 – 50	32.4 ± 7.8
Perceived Social Support Score (MSPSS)	24 – 84	58.6 ± 12.4
Depressive Symptoms Score (GDS-15)	0 – 14	6.1 ± 3.5

### 3.3 Univariate analysis of factors associated with foot self-care behavior

Categorical Variables (**Table 2**): Patients who had received foot care health education, those with a higher level of education, those with less severe ulcers (Wagner grades 1-2), those residing in urban areas, and those with comorbid hypertension demonstrated significantly higher scores in foot self-care behavior (P < 0.05). No statistically significant differences were found in the NAFF scores based on gender, marital status, smoking history, alcohol consumption history, hyperlipidemia, diabetic peripheral neuropathy, or different diabetes treatment regimen (P > 0.05).

**Table 2:** Univariate analysis of factors associated with foot self-care behavior (NAFF score) (N = 170).

Variable	Category	NAFF score (mean ± SD)	Statistic (t/F/r)	P-value
Age (years)	–	–	*r*=−0.08	0.298
Gender	Male	48.1 ± 10.5	*t*=−0.58	0.564
	Female	49.0 ± 9.7		
Marital status	Married	48.8 ± 10.1	*t* = 0.72	0.471
	Unmarried/Widowed	47.5 ± 10.5		
Education level	Junior high or below	46.2 ± 9.8	*F* = 8.24	<0.001
	High school	50.1 ± 9.5		
	College or above	53.4 ± 10.2		
Residence	Urban	49.8 ± 9.8	*t* = 2.45	0.015
	Rural	46.1 ± 10.5		
Diabetes duration (years)	–	–	*r*=−0.11	0.154
HbA1c (%)	–	–	*r*=−0.14	0.068
BMI (kg/m²)	–	–	*r* = 0.06	0.439
Smoking history	Yes	47.9 ± 10.4	*t*=−0.75	0.455
	No	49.0 ± 10.0		
Alcohol consumption	Yes	48.2 ± 10.1	*t*=−0.32	0.748
	No	48.7 ± 10.3		
Hypertension	Yes	49.6 ± 9.8	*t* = 2.18	0.031
	No	46.0 ± 10.6		
Hyperlipidemia	Yes	48.9 ± 10.0	*t* = 0.61	0.545
	No	48.0 ± 10.5		
Diabetic peripheral neuropathy	Yes	48.3 ± 10.1	*t*=−0.58	0.564
	No	49.4 ± 10.7		
Diabetes treatment regimen	No medication	47.1 ± 11.2	*F* = 1.54	0.206
	Oral medication only	49.2 ± 10.0		
	Insulin only	47.5 ± 10.3		
	Oral medication + Insulin	48.6 ± 10.1		
Wagner Grade	Grade 1-2	49.9 ± 9.6	*t* = 3.12	0.002
	Grade 3-4	45.7 ± 10.8		
Received foot care education	Yes	52.8 ± 8.7	*t* = 5.89	<0.001
	No	44.9 ± 9.9		
Foot care self-efficacy	–	–	*r* = 0.52	<0.001
Perceived social support	–	–	*r* = 0.38	<0.001
Depressive symptoms (GDS-15)	–	–	*r*=−0.35	<0.001

For categorical variables, independent samples t-test or one-way ANOVA was used; statistics presented as t-value or F-value. For continuous variables, Pearson correlation analysis was used; statistic presented as correlation coefficient r.

Continuous Variables: Pearson correlation analysis revealed that foot self-care behavior was significantly positively correlated with foot care self-efficacy (r = 0.52, P < 0.001) and perceived social support (r = 0.38, P < 0.001), and significantly negatively correlated with depression symptom scores (r = -0.35, P < 0.001). No significant correlations were observed between NAFF scores and age, duration of diabetes, body mass index (BMI), or HbA1c levels (P > 0.05). The detailed results of the univariate analysis are presented in **Table 2**.

### 3.4 *Post-hoc* subgroup analyses

In post−hoc subgroup analyses, the direction of associations between foot self−care behavior and the four identified factors (self−efficacy, health education, social support, depressive symptoms) remained consistent across age groups (<70 years vs. ≥70 years) and HbA1c levels (<7.5% vs. ≥7.5%). However, formal tests of interaction did not reach statistical significance for any of the stratifying variables (all *P* for interaction >0.05), likely due to the limited sample size within each stratum. These findings should be interpreted as exploratory and hypothesis−generating.

### 3.5 Multivariate linear regression analysis of factors associated with foot self-care behavior

Using the total score of the foot self-care behavior (NAFF score) as the dependent variable, variables that were statistically significant (P < 0.05) in the univariate analysis were included as independent variables in the multiple linear regression model. The assignment of independent variables is presented in **Table 3**. Prior to the regression analysis, assumption testing was conducted. The results indicated that the residuals followed a normal distribution with homoscedasticity, the tolerance for each independent variable was >0.6, and the VIF for each was <2.0, suggesting no issue of multicollinearity.

**Table 3:** Independent variables and their coding for multiple linear regression analysis.

Independent variable	Coding method
Education level	Junior high or below = 1, High school = 2, College or above = 3
Residence	Urban = 1, Rural = 0
Hypertension	Yes = 1, No = 0
Wagner Grade	Grade 1-2 = 1, Grade 3-4 = 2
Received foot care health education	Yes = 1, No = 0
Foot care self-efficacy	Continuous (original score)
Perceived social support	Continuous (original score)
Depressive symptoms	Continuous (original score)

The regression model was statistically significant (F = 18.74, P < 0.001), explaining 47.2% of the variance in foot self-care behavior (adjusted R² = 0.472). The results revealed that foot care self-efficacy (β = 0.41, P < 0.001), having been subjected to foot care health education (β = 0.25, P = 0.001), perceived social support (β = 0.19, P = 0.008), and depression symptoms (β = −0.17, P = 0.021) were factors associated with foot self-care behavior in elderly patients with recurrent diabetic foot. Detailed data are shown in **Table 4**.

**Table 4:** Multiple linear regression analysis of factors associated with foot self-care behavior.

Variable	Unstandardized coefficient (B)	Standard error (SE)	Standardized coefficient (*β*)	t-value	P-value
(Constant)	12.45	4.21	–	2.96	0.004
Foot care self-efficacy	0.54	0.10	0.41	5.62	<0.001
Received health education	5.10	1.48	0.25	3.45	0.001
Perceived social support	0.16	0.06	0.19	2.71	0.008
Depressive symptoms	-0.50	0.21	-0.17	-2.33	0.021
Education level	1.24	0.98	0.09	1.27	0.207
Residence (Urban)	2.10	1.56	0.10	1.35	0.180
Hypertension (Yes)	1.56	1.62	0.07	0.96	0.337
Wagner Grade (Grade 3-4)	-1.87	1.56	-0.08	-1.20	0.232

Model summary: *R* = 0.698, *R*^2^ = 0.487, Adjusted *R*^2^ = 0.472, F = 18.74, P<0.001.

## 4 Discussion

This study is the first to focus on the specific and vulnerable population of elderly patients with recurrent DFU, aiming to investigate the factors influencing their foot self-care behaviors. The results indicate that the overall level of foot self-care in this population is moderate, with considerable room for improvement. Multiple linear regression analysis ultimately identified four factors associated with foot self-care behavior: foot care self-efficacy, foot care health education, perceived social support, and depressive symptoms. Together, these four factors accounted for 47.2% of the variance in foot self-care behavior. These findings provide profound clinical guidance for developing targeted interventions to enhance self-care levels and reduce the risk of ulcer recurrence.

Foot care self−efficacy emerged as the strongest associated factor. This result aligns with prior evidence linking higher self−efficacy to better foot self−care in various diabetic populations (6, 22, 23). For example, Sezgunsay et al. (22) reported a comparable positive correlation between self−efficacy and foot care behavior in Turkish patients with DFU. A plausible explanation is that individuals who believe they can successfully perform foot care tasks are more likely to translate knowledge into consistent action. However, it is noteworthy that older adults with recurrent DFU may face a distinctive erosion of self−efficacy due to repeated treatment failures and ulcer relapses—a dynamic less pronounced in first−episode or ulcer−free high−risk populations (6). From our perspective, this underscores the importance of not merely imparting foot care knowledge, but deliberately rebuilding self−confidence through techniques such as mastery experiences, role modeling, and individualized encouragement, which may be especially critical in this recurrent−ulcer subgroup.

Receipt of foot care health education was another factor significantly associated with better self−care. A systematic review incorporating 26 studies demonstrated that diabetic foot education improved the knowledge level and behavioral compliance of patients with diabetes (24). Thomson et al. found that 39% of elderly patients with diabetes were unable to touch their toes, and only 14% of elderly patients were responsive to plantar lesions. This indicates that in the absence of supportive interventions, foot care education alone may not be sufficient to effectively reduce the incidence of foot problems in elderly diabetic patients (25). Notably, the target population of this study was older adults, who may experience issues such as cognitive decline and reduced learning capacity. We therefore argue that effective education must be multimodal, repetitive, and integrated with practical skill-building and caregiver involvement to overcome physical, sensory, and cognitive barriers.

Perceived social support was independently and positively associated with foot self−care. This is consistent with studies showing that patients who live with family or perceive stronger support exhibit better diabetic foot care (26, 27). Social support likely operates through both instrumental pathways and emotional pathways. In our view, the importance of social support is magnified in older adults with recurrent DFU, who frequently contend with multimorbidity, polypharmacy, and functional decline. Clinically, actively involving family members and primary caregivers in education and care plans represents a logical and feasible strategy to bolster the patient’s self−care capacity.

Depressive symptoms were negatively associated with foot self−care behavior. This finding corroborates the well−documented adverse impact of depression on diabetes self−care, including foot care (28, 29). Gonzalez et al. (30) prospectively showed that higher baseline depressive symptoms predicted poorer foot care adherence at follow−up. The recurrence of DFU, accompanied by chronic pain, limited mobility and fear of amputation, may create a vicious cycle: depressive symptoms reduce patients’ motivation for self-care, which in turn may lead to ulcer recurrence. It should be noted that our study used the GDS−15, a scale validated specifically for geriatric populations, whereas other studies have employed different instruments such as Harvard Department of Psychiatry/National Depression Screening Day Scale (HANDS) (31) and the 10-item Center for Epidemiologic Studies Depression Scale (CESD-10) (32); this methodological variation may partly account for subtle differences in reported effect sizes across the literature. Our results support the integration of routine depression screening into DFU care and suggest that even subclinical depressive symptoms warrant clinical attention to mitigate their potential impact on self−care behaviors.

Collectively, these findings suggest that clinical management of recurrent DFU should extend beyond localized wound care to incorporate structured psychosocial support. Interventions that simultaneously enhance self-efficacy, deliver tailored education, engage caregivers, and routinely screen for depressive symptoms may contribute to improved self-care behaviors. However, given the cross-sectional design, these associations should be viewed as hypothesis-generating rather than prescriptive. Longitudinal and interventional trials are needed to determine whether targeting these factors causally improves clinical outcomes and reduces recurrence rates.

## 5 Limitations

This study has several limitations. First, the cross-sectional design limits causal inference. Second, self-reported data may be subject to recall and social desirability bias. Third, the use of purposive sampling from only three medical centers in China may introduce selection bias and limit generalizability. Fourth, face-to-face interviews, while necessary for this population, could introduce interviewer bias despite standardized procedures. Fifth, although *post-hoc* subgroup analyses were conducted, the relatively modest sample size limited the statistical power for formal interaction tests, and thus potential effect modification by age or HbA1c could not be reliably examined. Finally, unmeasured confounding variables, such as cognitive function and health literacy levels, may have influenced the observed associations. Future research should employ longitudinal, multicenter designs and include detailed cognitive assessments.

## 6 Conclusion

In summary, this study found that foot self-care behaviors among elderly patients with recurrent DFU are at a moderate level and are significantly associated with multiple modifiable psychosocial factors, including self-efficacy, health education, social support, and depressive symptoms. Clinical interventions should adopt a multidimensional approach, focusing on enhancing self-confidence, providing individualized education, and mobilizing support systems. Future longitudinal studies are needed to determine whether targeting these factors can effectively improve self-care and reduce ulcer recurrence in this high-risk population.

## Data Availability

The original contributions presented in the study are included in the article/supplementary material. Further inquiries can be directed to the corresponding authors.
